# Predictors of hysterectomy among married women 15–49 years in India

**DOI:** 10.1186/s12978-017-0445-8

**Published:** 2018-01-05

**Authors:** Ranjan Kumar Prusty, Chetan Choithani, Shiv Dutt Gupta

**Affiliations:** IIHMR University, 1, Prabhu Dayal Marg, Sanganer Airport, Jaipur, 302 029 India

**Keywords:** Hysterectomy, Socio-economic status, Household health insurance, India, Gynechological ailments

## Abstract

**Background:**

In India, community based studies and media reports indicate a surge in the number of young women undergoing hysterectomy in the past few years. This has led to suspicion on the misuse of procedure, and intense debates on its potential ill health-effects on young women. However, there are no population-based studies that provide insights into hysterectomy prevalence and its determinants at the national level.

**Data and methods:**

This study used data from India’s District Level Household Survey that involved a sample of 3, 16,361 married women in the age group of 15–49 years spread across 21 States and Union Territories of India. Bivariate and multivariate regression analysis was performed to estimate hysterectomy prevalence and identify its predictors.

**Results:**

The study estimated hysterectomy prevalence of 17 per 1000 ever married women. The number of women undergoing hysterectomy ranged from 2 to 63/1000 across different states. A little more than one-third of women who had undergone hysterectomy were under the age of 40 years. The proportion of women below 40 years of age who had had hysterectomy was much higher in southern states of Andhra Pradesh (42%) and Telangana (47%). The likelihood of hysterectomy was higher among women belonging to households with health insurance (OR: 1.88, CI: 1.77–2.00) and women who were sterilized (OR 1.55; CI 1.45–1.67) than uninsured and unsterilized women, and lower among women with education level of matriculation and above (OR 0.47; CI 0.42–0.50) than those with no and/or low education.

**Conclusions:**

A sizable proportion of young women undergoing hysterectomy in India may have severe ill-health effects on their physical, reproductive and socio-psycho health. As women with low or no education are also more prone to hysterectomy, providing more information and education to them on the possible after-effects of hysterectomy and alternative options will enable them to make more informed choices.

## Plain English summary

Hysterectomy is a leading reason for non-obstetric surgery in many countries. However, the procedure is also found to have adverse health effects on women’s physical and socio-psycho health, particularly on pre-menopausal, young women. In India, in recent years there appears to be a surge in hysterectomy cases involving young women. This has led to suspicion on the misuse of procedure. However, there are no population-based studies that provide insights into hysterectomy prevalence and its determinants at the national level. This study makes this contribution using data from District-Level Household Survey-4 which covered a sample of 3, 16,361 married women aged 15–49 years from 21 states and union territories of India.

Our findings show that the current median age of the women who had undergone hysterectomy was 42 years. One-third of hysterectomized women were below the age of 40 years, and this proportion was higher in Southern Indian states of Andhra Pradesh (42%) and Telangana (47%). Statistical analysis show that hysterectomy is more common among women who had no and/or low education and those from households with health insurance. These findings indicate a need for counselling and education of lowly educated young women on alternative options. Secondly, it appears that health insurance is possibly also leading to unnecessary hysterotomies among young women which warrants a need for better designing of insurance systems. We also note that reasons for hysterectomy are however complex, and there is thus a need for more robust data systems to understand the determinants of hysterectomy more fully.

## Article summary

### Strength

- This paper draws on the data from a large-scale survey on reproductive and child health in India and thus the results of this study have wider relevance.

### Limitation

- The survey did not collect information on history of hysterectomy and only self-reported prevalence of hysterectomy was considered in the survey. Additionally, it provided no information on age at hysterectomy, total costs incurred and sources of financing for the procedure, and problems faced by women after hysterectomy.

## Background

Hysterectomy, the surgical removal of uterus, is the second most frequently performed non-obstetric surgery after cesarean section in many parts of the world [1–5]. Moreover, to reduce the future risk of ovarian cancer, prophylactic oophorectomy which involves removal of ovaries is often recommended simultaneously with hysterectomy [6]. Common medical indications of hysterectomy include gynecological ailments such as fibroids, dysfunctional uterine bleeding, uterine prolapse [7]. In other words, most hysterectomies are performed for benign gynecological reasons. For instance, in the United States, among women aged 15 years and above who underwent hysterectomy during 2000–04, *uterine leiomyoma* was the most common hysterectomy indication accounting for nearly 41% of all hysterectomies followed by *endometriosis* (17.7%) and *uterine prolapse* (14.5%), while *uterine cancer* accounted for 9.2% of all cases [3].

The surgical removal of uteri and ovaries can have important bearing on women’s physical and psychosocial health. Research shows both positive and negative after-effects. On the one hand, by relieving suffering from gynecological ailments such as abnormal bleeding and pelvic pain, hysterectomy is found to lead to decreased anxiety and depression among women and thereby improvement in their quality of life, particularly 6 to 12 months after the surgery [8–10]. On the other hand, research also shows several adverse effects of hysterotomy such as urinary incontinence [11], sexual dysfunction [12–14], late medical problems such as backache and weakness [15] and earlier onset of menopause [16]. Moreover, concomitant oophorectomy increases women’s risk to osteoporosis and coronary heart disease and thus poses excess mortality risk [6]. Because of these health impacts of hysterectomy and the fact that most surgeries relive women only of benign gynecological issues, many health professionals argue for alternative treatments, and that hysterectomy should be resorted to only in the case of life-threatening diseases [17, 18].

The incidence and prevalence of hysterectomy varies widely across different geographic settings due to variations in uterine pathology, providers and patient factors and socio-cultural reasons [4, 19, 20]. Because the available research on hysterectomy is largely based on inpatient facilities and community-based studies, differences in sample populations and methodologies cloud the global comparison of hysterectomy rates. Nonetheless, research shows that hysterectomy rates are much higher in the developed countries compared to low-income countries [20]. In US, hysterectomy prevalence is estimated to be 26.4% [21]. Community based studies from Australia show hysterectomy prevalence ranging from 16.9% among women aged 18–69 years [22] to 22% among women in the ages of 45–50 years [1]. More recent research shows decline in hysterectomy rates in many parts of the developed world, as less-invasive alternatives to hysterectomy such as endometrial ablation and uterine artery embolization become more widely available. In US and Canada, for instance, hysterectomy prevalence has declined in recent years [3, 4]. On the other hand, hysterectomy appears to be on rise in some developing countries [23, 24].

In India, hysterectomy has received increased attention in health policy debates in the past few years. The trigger for increased focus is provided by a series of media reports that have highlighted an unusual surge in the number of women undergoing hysterectomy in many parts of the country, with a significant number of cases involving young and pre-menopausal women from poor families [25–29]. This rising number of young women undergoing hysterectomy has raised suspicions about unscrupulous practices on the part of health care providers for profit reasons. Research evidence from recent studies on hospital facilities and insurance provide some credence to this ‘malpractice-for-profit’ hypothesis. A study by Kameswari and Vinjamuri (2013) involving a sample of 171 women in Andhra Pradesh found during 2008–2010, 60% of hysterectomies were carried out on women aged under 30 and that 95% of the operations were done in private hospitals; the hospital discharge summaries of these operations were mostly blank, with no information about the procedure or follow-up instructions [30]. Findings of another study in a low-income setting in Ahmedabad district of Gujarat showed that hysterectomy was a leading reason for hospitalization and insurance claims [20]. Alarmed by this, in 2013, in response to a public interest litigation filed by Human Rights Law Network, the Indian Supreme Court issued notices to state governments of Bihar, Rajasthan and Chhattisgarh to check this malpractice. States such as Andhra Pradesh have imposed restrictions on private hospitals to perform hysterectomy under public insurance schemes [23, 31].

Other assessments however suggest that the reasons for hysterectomy are complex. Based on research on women from low-income families in Ahmedabad, Gujarat, Desai (2016) warns against this sole narrative of women as ‘passive victims’, and argues that choice to undergo hysterectomy also reflects ‘pragmatic agency’ of women [23]. She found that compelled to “earn and care for their families, women balanced their medical options with social responsibilities. In this way, bio-medicine and its negotiations were enacted in spheres of work and family, beyond the provider-patient interaction” (p. 16). Another study from Maharashtra showed that besides prescription by health care providers, other reasons why women opted for hysterectomy included lack of faith in alternative treatments to hysterectomy, fear of cancer and its future consequences, failure of ongoing medical treatment and practical difficulties in living with reproductive health problems [32].

The significance of these findings notwithstanding, a comprehensive assessment of hysterectomy prevalence, and its correlates at the national level is missing. The limited evidence on hysterectomy in India comes from the community studies, and to our knowledge there is a no population-based study on the subject matter. Using the data from the fourth round of District Level Household and Facility survey (DLHS), this paper estimates the prevalence of hysterectomy, identifies hysterectomy patterns and examines the underlying socio-economic determinants of hysterectomy in India.

The layout of this paper is as follows. In the next section, we describe the Methods and Data source used in this paper. Results section presents the finding on the patterns and predictors of hysterectomy in India. This is followed by a discussion of these results in the Discussion section. Conclusion section summarises the main results. Last section highlights the policy relevance of the key research findings.

## Methods

### Data source

The present study uses secondary data from the fourth round of DLHS, conducted in 2012–13. DLHS-4 is a large-scale sample survey focused on a wide range of reproductive and child health (RCH) issues. This survey was commissioned by the Ministry of Health and Family Welfare, Government of India (GoI). The survey was carried out in 14 states and seven union territories of India. These states are: Andaman & Nicobar Islands, Andhra Pradesh, Arunachal Pradesh, Chandigarh, Goa, Haryana, Himachal Pradesh, Karnataka, Kerala, Maharashtra, Manipur, Meghalaya, Mizoram, Nagaland, Puducherry, Punjab, Sikkim, Tamil Nadu, Telangana, Tripura, West Bengal.

 (Eight states – Bihar, Jharkhand, Madhya Pradesh, Chhattisgarh, Orissa, Rajasthan, Uttar Pradesh and Uttarakhand – forming part of Empowered Action Group (EAG) States were excluded. These EAG states are worst performing states of the country lagging far behind than other Indian states on social, demographic and health indicators. Additionally, the state of Gujarat and Assam were also left out from the survey.) We refer to these 21 States and Union Territories as Non-EAG Indian States. The survey collected information on maternal and child health, family planning, and other reproductive health indicators from 3,16,361 women in the reproductive ages of between 15 and 49 years from a total of 3,78,487 households from these states. A multi-stage stratified systematic sampling design was adopted which yielded state-representative samples after applying sampling weights to control for complex survey design [33]. For the analysis in this paper, we have pooled data from these 21 States and Union Territories.

In DLHS-4, information was collected using separate questionnaires for household, ever married women, unmarried women, villages, and health facility using Computer Assisted Personal Interviews (CAPI). Information on household members and socio-economic conditions of the household like caste, religion, and information about household wealth was collected through household questionnaire. The ever-married women questionnaire covered information on different components of maternal and child health, including pregnancy, child birth, reproductive morbidities, immunization of mothers and children, and access and availability of maternal and child health care. Among unmarried women, questionnaire included questions related to reproductive health of unmarried women aged 15–24 years. The village and health facility questionnaire covered availability of infrastructure, human resources at the health facilities and availability of basic resources in the respective villages and health facilities where the survey was conducted.

The survey adhered to strict protocols to ensure high data quality. Rigorous training of trainers was conducted for three weeks in use of CAPI and data collection methods as well as the technical aspects of questionnaires by the International Institute for Population Sciences, GoI’s nodal agency in-charge of this survey. The trainers subsequently trained the data collectors for three weeks to ensure that they are acquainted with CAPI and the questionnaires, asking questions and recording the responses. During the survey, a rigorous monitoring protocol was followed that involved spot checks, back checks, and system generated field check tables to ensure high data quality.

### Data analysis

For this paper, the data analysis was conducted using IBM SPSS 20 package. The details of dependent and independent variables are given below:

- *Dependent variable:* Women were asked about their current mensuration status. Among those women who reported not menstruating were asked if they had been operated for hysterectomy. This information was used to estimate the prevalence of hysterectomies among women aged 15–49 years.
- *Independent variables:* Socio-economic and demographic variables like age of women, caste, religion, parity, place of residence, working status, household wealth, household with health insurance, women sterilization and education of women were used as independent variables.

In large-scale household surveys, wealth index is increasingly used to measure household economic status. This index is computed using Principal Component Analysis (PCA) based on an arbitrary scoring of household economic indicators such as housing quality, household amenities, size of landholding, and consumer durables etc. This index is then divided into five quintiles which include poorest, poorer, middle, richer, and richest [34–36]. In this paper, we also computed wealth index to assess the economic status of household sample women belonged to. For our analysis, we created three wealth categories instead of five: we grouped poorest and poorer as ‘Poor’, middle as ‘Middle’ and richer and richest as ‘Rich’.

Univariate and bivariate analyses were carried out to assess socio-economic differentials, which were used as predictors. Multivariate logistic regression was used to estimate the risk of hysterectomies using Odds Ratio (OR). The following logit was used to estimate the risk of hysterectomy.

$$ \mathrm{In}\left(\frac{{\mathrm{p}}_{\mathrm{i}}}{1\hbox{-} {\mathrm{p}}_{\mathrm{i}}}\right)={\upbeta}_0+{\upbeta}_{1{\mathrm{x}}_1}+\cdots +{\upbeta}_{{\mathrm{Mx}}_{\mathrm{m},\mathrm{i}}} $$

Where β_0_,...,β_n_ are regression coefficients indicating the relative effect of an explanatory variable on the outcome which is hysterectomy in this case.

## Results

### Hysterectomy prevalence and spatial patterns

**Fig. 1:**
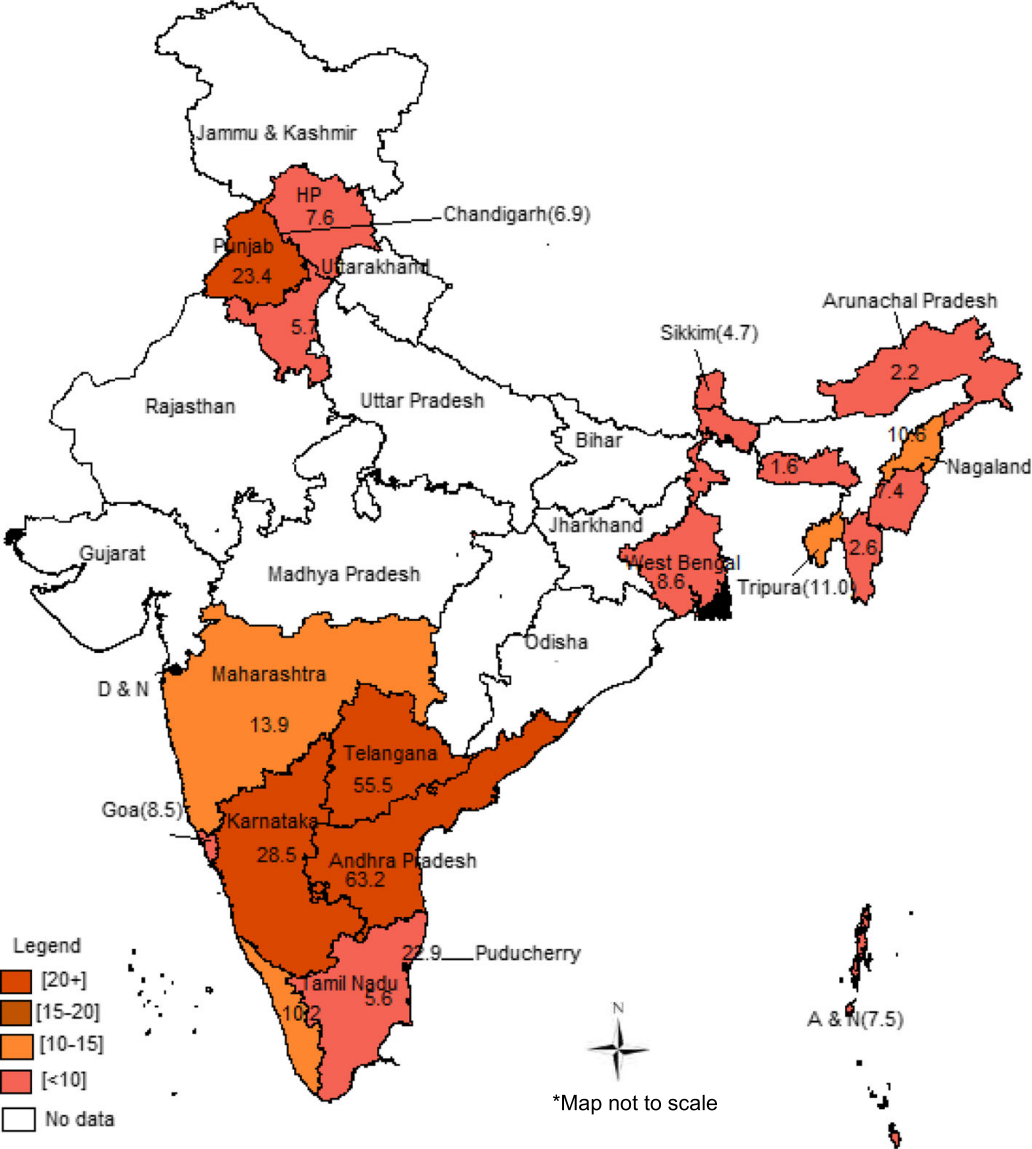
Spatial patterns of hysterectomy among women 15–49 years (‘000) in India, 2012-13. Source: Authors’ work based on DLHS-4 data. HP = Himachal Pradesh; A & N = Andaman & Nicobar; D & N = Dadra and Nagar Haveli

The average prevalence rate of hysterectomy was estimated to be 17/1000 among ever married women in the ages of 15–49 years. As many as 5567 women out of the total 3, 16, 361 reported having undergone hysterectomy. There were wide variations in the prevalence rates across the different states and union territories in India, ranging from 2/1000 to 63/1000 women. Among the large Indian states, the lowest prevalence rates of hysterectomy were reported in the states of Tamil Nadu and Haryana – nearly 6/1000 women in both states. On the other hand, the state of Andhra Pradesh had the highest prevalence rate of hysterectomy (63/1000 women), followed by Telangana (55/1000), Karnataka (29/1000) and Punjab (23/1000) (Fig. 1)

.

### Socio-economic differential and predictors of hysterectomy

#### Current age

**Fig. 2:**
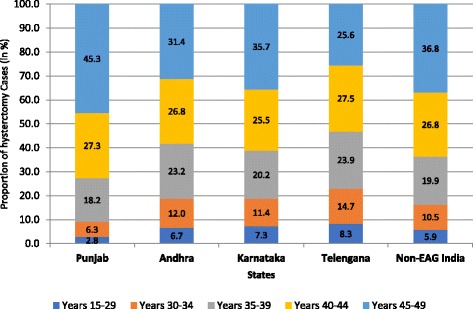
Proportion of women who underwent hysterctomy by the age of women in selected Indian states, 2012–13

The current median age of the women undergone hysterectomy was 42 years in all the states covered in the survey. It is important to note that many women reported undergoing the surgeries at younger ages: more than one-third (36%) of all the women who got their hysterectomy done did so before reaching 40 years of age. Furthermore, the high hysterectomy prevalence states of Andhra Pradesh and Telangana had a much higher proportion of women under 40 years of age who had hysterectomy, 42% and 47% respectively (Fig. 2). It is important to note that this paper utilized the information on current age of women during the survey, and not age at which women underwent hysterectomy. The implication of this is that proportion of young women undergoing hysterectomy will likely be higher.

Age was a significant predictor of hysterectomy among women: the risk of hysterectomy increased with the increasing age. This is consistent with findings from studies from other parts of the world [1, 4]. It was also an independent predictor of hysterectomy when adjusted for the other factors. The odds ratio, obtained through multivariate logistic regression analysis, was 5.89 (CI 5.17–6.76) in 40–44 years which increased to 8.60 (CI 7.57–9.86) for women aged 45–49 years.

#### Parity

Results showed that there was a greater proportion of women undergoing hysterectomy with higher parity (Table 1). The unadjusted estimates showed a five times higher risk of hysterectomy (OR 5.24; CI 4.47–6.14) among women who had two or more children. However, when adjusted for other factors, women with two or more children had only 74% higher risk of hysterectomy than those with no children; this was statistically significant (OR 1.74; CI 1.41–2.14). Like age, parity was also an independent and significant predictor.

**Table 1:** Socio-economic differentials in prevalence of hysterectomy among married women aged 15–49 years in selected states of India, 2012–13

Background Characteristics	Non-EAG (Pooled)	
	hys/1000	N
Child Ever Born		
0	4.4	37,077
1	7.6	64,320
2	18.6	113,916
3	25.7	61,154
4+	27.6	43,228
Religion		
Hindu	18.7	224,788
Muslim	14.9	29,021
Christian	8.3	32,331
Sikh	24.9	21,083
Others	7.3	12,472
Residence		
Rural	18.0	190,812
Urban	16.2	128,883
Education		
Nonliterate	25.5	72,583
Less than 5	33.0	33,251
6–9 years	15.1	94,869
10 or more year	9.7	118,992
Caste of Household		
SC & ST	13.3	126,488
OBC	20.8	108,268
Other	20.2	68,819
Working		
Not working	14.7	248,913
Working	26.3	70,549
Household with Insurance		
No	14.1	242,443
Yes	28.1	72,571
Wealth Quantile		
Poorest	14.5	62,967
Poorer	16.4	63,198
Middle	19.2	63,091
Richer	19.2	63,077
Richest	17.4	63,083

*SC* Scheduled Caste, *ST* Scheduled tribes, *OBC* Other backward Classes, *N* Sample Size

#### Religion and caste

We also found differentials in hysterectomy prevalence by religion and caste. The proportion of women going for hysterectomies was higher among Sikh women (25/1000), followed by Hindus (19/1000), Muslims (15/1000) and Christian (8/1000). The multivariate results revealed that Sikh women were significantly more likely (OR 1.46; CI 1.32–1.63) and Muslim (OR 0.89; CI: 0.79–1.00) and Christian (OR 0.61; CI 0.53–0.71) women were significantly less likely to choose hysterectomies compared to Hindu women. Analysis by caste showed that the women belonging to upper caste groups (labelled as ‘Others’ in DLHS-4) and Other Backward Classes (OBCs) had significantly higher odds of going for hysterectomies than the women from scheduled castes and tribes (Table 2).

**Table 2:** Logistic regression showing bivariate and multivariate odds ratio of married women (15–49 years) going for hysterectomy in Non-EAG states of India

Background variables	Odds Ratio (95%-CI)	Adjusted Odds Ratio (95%-CI)
Age of women		
< 30	1.00	1.00
30–34	3.56 (3.11–4.09)***	2.42 (2.09–2.81)***
35–39	6.63 (5.85–7.52)***	3.86 (3.37–4.43)***
40–44	10.94 (9.68–12.36)***	5.89 (5.17–6.76)***
45–49	16.39 (14.55–18.47)***	8.60 (7.57–9.86)***
Child Ever Born		
0	1.00	1.00
1	1.79 (1.49–2.14)***	1.29 (1.03–1.61)*
2+	5.24 (4.47–6.14)***	1.74 (1.41–2.14)***
Residence		
Rural	1.00	1.00
Urban	0.99 (0.94–1.05)	0.99 (0.93–1.06)
Caste of Household		
SC & ST	1.00	1.00
OBC	1.58 (1.48–1.69)***	1.39 (1.29–1.50)***
Other	1.56 (1.45–1.68)***	1.49 (1.36–1.61)***
Religion		
Hindu	1.00	1.00
Muslim	0.82 (0.75–0.91)***	0.89 (0.79–1.00)*
Christian	0.41 (0.36–0.47)***	0.61 (0.53–0.71)***
Sikh	1.36 (1.24–1.49)***	1.46 (1.32–1.63)***
Others	0.37 (0.30–0.45)***	0.46 (0.35–0.59)***
Working		
Not working	1.00	1.00
Working	1.77 (1.67–1.87)***	1.47 (1.38–1.57)***
Household with Insurance		
No	1.00	1.00
Yes	1.99 (1.88–2.10)***	1.88 (1.77–2.00)***
Women Sterilized		
No	1.00	1.00
Yes	3.06 (2.88–3.26)***	1.55 (1.45–1.67)***
Education		
No/Primary school	1.00	1.00
Secondary	0.54 (0.51–0.57)***	0.66 (0.61–0.71)***
Matriculation or higher	0.35 (0.33–0.38)***	0.47 (0.42–0.50)***
Wealth Index		
Poor	1.00	1.00
Middle	1.26 (1.17–1.35)***	1.28 (1.18–1.40)***
Rich	1.25 (1.17–1.33)***	1.48 (1.36–1.60)***

a) The first categories of the variables are reference categories; b) SC: Scheduled Caste, ST: Scheduled tribes, OBC: Other backward Classes; c) first two quintiles include ‘Poor’, third is ‘Middle’ and fourth & fifth are grouped as ‘Rich’; d) women having no schooling or less than 5 years of schooling are considered as <5 years of schooling; e) statistical significance levels: *P < 0.05, **P < 0.01, ***P < 0.001

#### Working and education status of women

**Fig. 3:**
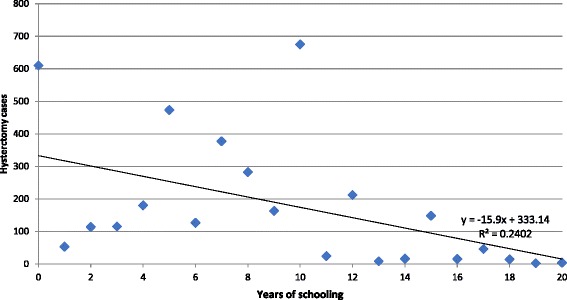
Hysterectomy by years of schooling of women in India, 2012–13

Education also determined whether or not a woman was likely to undergo the surgery. The level of education was found to exert negative influence on hysterectomy: as women’s years of schooling increased, there was a decline in hysterectomy rate (Fig. 3). The multivariate analysis showed women who had education levels of matriculation and above were 53% less likely to undergo hysterectomy than women with no or primary school education.

#### Household wealth

As noted above, we used household wealth as a proxy indicator of economic status. An asset based wealth quintile was constructed using principal component analysis (PCA). The women belonging to rich households were more likely to choose for hysterectomies (OR 1.48; CI 1.36–1.60) than the women from the poor households.

#### Health insurance

Our analysis also found that the proportion of women undergoing hysterectomy was higher among household with health insurance compared to those without. The women in households with health insurance were almost two times more likely to choose hysterectomies than women whose household were not covered with health insurance. In the high hysterectomy prevalence southern states, likelihood of women from household with insurance undergoing hysterectomies vis-à-vis those without was highest in Andhra Pradesh (OR: 1.46, *P* < 0.001), followed by Telangana (OR: 1.31, *P* < 0.01) and Karnataka (OR: 1.22, *P* < 0.01).

#### Sterilization

Hysterectomy was found to be high among women who were sterilized (tubectomy) than those not sterilized. The women who were sterilized were 55% more likely to choose hysterectomy (OR 1.55; CI 1.45–1.67) than those not sterilized after adjusting for other socio-economic factors. Previous studies have found that types of tubal ligation may be associated with a later risk of menstrual disorders and hysterectomy, particularly in women sterilized at a young age [37, 38]. Our findings further corroborate the findings of earlier studies on the association between sterilization and hysterectomy.

## Discussion

The overall prevalence of hysterectomy across the different states covered in DLHS-4 varied from 2 to 63/1000 women. Southern states stand out for considerably higher prevalence of hysterotomy, with Punjab in north India being the exception. In particular the states of Andhra Pradesh and Telangana (which were one state until recently) appear to be the hotspots of hysterectomy. Indeed these states have been the focus of debate on unnecessary and forced hysterectomies in India, particularly involving young, pre-menopausal women from the poor socio-economic backgrounds. The existing research shows that in some parts of these states such as Medak district in Andhra Pradesh, poor rural women are coerced into hysterectomy even for routine gynecological issues, such as abdominal pain and white discharge, by health care providers often for profit motives and without offering alternatives [39]. In villages such as Kannaram village of Medak, most women including those as young as 20 years have had their uteri removed leading a national daily to describe the health service providers in terms as scathing as ‘the uterus snatchers of Andhra [Pradesh]’ [40].

Most of these hysterectomy surgeries are done at private hospitals [27, 30]. These hysterotomies are attributed to the State Government’s Aarogyashri health insurance scheme. Initiated in 2007, Aarogyashri provides health care coverage of upto 1.5 lakhs (approximately US$ 2500) to individuals from poor families, including costs incurred on hospitalization and surgery at private hospitals [39]. Data show that during 2008–09 and 2009–10, the total number of hysterectomies performed in (united) Andhra Pradesh under Aarogyashri were 10, 334 and 12, 212 respectively. In response to this, in 2010 the state government initiated restrictions on private hospitals to claim benefits under this health insurance scheme for hysterectomy which led to number of such surgeries dipping to 6189 during 2010–11, and further to 4943 during 2011–12 [31]. The linkage between health insurance and hysterectomy is not just restricted to Andhra Pradesh and Telangana. Studies conducted in Gujarat also find hysterectomy to be a leading reason of health insurance claims [20, 41]. Our analysis that included 21 Indian states and Union Territories also found household health insurance to be a crucial factor that influenced hysterectomy in that women belonging to households covered under insurance sought hysterectomy more than uninsured women. Research shows that health insurance is an important means which enables poor households to seek health care, and protects them from catastrophic health expenses [42]. However, misuse of health insurance in the case of hysterectomy warrants a need to revisit the design of insurance schemes in order to check unnecessary hysterectomies.

It is important to note that profit-motive may not always be the most important factor, let alone the sole reason, why providers recommend and women choose hysterectomy for routine gynecological morbidities in India. The ‘normalization of hysterectomy’ is widespread in many developed countries with considerably better health systems and checks in place. In US, for instance, there are potential alternative treatment options available for nearly 90% of the total 6,00,000 annual hysterectomy surgeries [18]. It appears that health providers’ training and patients’ preferences and fears also operate to influence hysterectomy rates. Research by Desai et al. (2017) on hysterectomy among poor Gujarati women working in informal sector showed that while many women viewed uterus productive solely for the reproductive function it served and thus sought hysterectomy when faced with benign gynecological issues that affected their day-to-day lives, health care providers often favoured hysterectomy keeping in view the life circumstances and socio-economic realities that characterized the life worlds of these women [20]. To quote one of the health care providers belonging to a private hospital in their study: “Generally it is better to do hysterectomy [than other procedures] because the [rural] patient is not going to take continuous treatment and medical management gets costly … and we can't say how much that will help anyhow. So it's better that they have a hysterectomy. And ultimately the problem gets solved” (p. 16). These complex dynamics of hysterectomy decisions-making on the part of both providers and patients warrant a need for more research on patient-provider factors that influence hysterectomy in India.

Our findings also show that many of the hysterectomies involve young women. As noted earlier, more than one-third (36%) of the women who underwent hysterectomy were less than 40 years of age. Cross-country evidence shows that hysterectomy is more commonly sought by women at the later stages of their lives for various gynecologic ailments [3, 4, 21]. For instance, in US, mean age of hysterectomy is 50.76 years [21]. Given the adverse health effects of the procedure, as noted above, hysterectomy at younger ages may lead to earlier onset of poor physical and reproductive health outcomes [43–46]. Concurrent bilateral oophorectomy which is frequently performed with hysterectomy is generally recommended for women over the age of 40 years. This is because it induces immediate surgical menopause and ovaries perform a useful function of generating estrogen which reduces the risk of osteoporosis and coronary heart disease [6]. Studies show that women who experience premature menopause (before age 40 years) or early menopause (between ages 40 and 45 years) have increased risk of excess mortality, cardiovascular, neurological, and psychiatric diseases, osteoporosis, and other sequelae [6, 45, 47]. Furthermore, surgically induced menopause as a result of prophylactic oophorectomy is also found to increase the risk of cognitive impairment or dementia among women, with this risk being higher among younger women, and negatively affects the quality of life [44]. A comparative study of surgically or naturally menopausal women found that those who had hysterectomy with bilateral oophorectomy were more likely to have low sexual desire, less likely to be sexually active, and more likely to be dissatisfied with their sex life and partner relationships [48].

In addition, we found that sterilized women (tubal ligation) were more likely to undergo hysterectomy than women who were not sterilized. Other studies have also shown similar association between sterilization and hysterectomy, as tubal ligation has been associated with higher risk of menstrual disorders and gynecological ailments [37, 38, 49–51]. DLHS-4 data show that a sizable proportion of women also underwent sterilization at young ages (less than 25 years) in high hysterectomy prevalence states of Andhra Pradesh (39%), Telangana (26%) and Karnataka (58%). The median age of sterilization in Karnataka, Andhra Pradesh and Telangana was 24, 25 and 27 years, respectively [33]. Research based on nationwide National Family Health Survey-3 data reveal that early age of sterilization often also leads to many women regretting their decisions later. At all-India level, 5% of all women who underwent sterilization regretted their decision later, and women who were sterilized at 30 years or later had lesser likelihood of ‘sterilization regret’ than those sterilized before age 25 [52]. Findings from a community based study in India involving sample of 1000 women with hysterectomy prevalence of 7% (70 women) showed that post hysterectomy many women reported late medical problems such as backache, vaginal discharge, weakness and incontinence [15]. Interpreting the findings of these studies on sterilization and hysterectomy together, young women undergoing hysterectomy post sterilization may also have ‘hysterectomy regret’ because of these issues, with potentially negative impact on their mental wellbeing and quality of life. This warrants further research.

Lastly, schooling and household wealth were found to have significant influence on hysterectomy. Our result showed women who had higher education were less likely to go for hysterectomy than women who had no education or attended school only up to primary level. On the other hand, women from economically better-off households were more likely to choose this surgical procedure than those from poor households. This indicates that women with resources but less education are perhaps more prone to hysterectomy and there is need for better education among them on alternative options.

## Conclusion

This study has attempted to analyse hysterectomy prevalence and its socio-economic determinants using the data covering 21 states and union-territories of India. Our findings revealed that age, prior sterilization, household insurance status influence the propensity of hysterectomy. Although evidence on the health after-effects of hysterectomy is mixed, research shows that hysterectomy at an early age has severe ill-effects on women’s physical, mental and social wellbeing including incontinence [11], sexual dysfunction [12–14], and earlier onset of menopause with increased risks of cardiovascular diseases [6, 16]. In India, the research and debate on hysterectomies for benign reproductive health ailments has tended to focus invariably more on health providers’ (mal)practices. Barring one notable exception of a study by Desai et al. (2017), a holistic understanding of health and socio-cultural contexts that guide patients’ and providers’ preferences for hysterectomy against alternative options is sorely lacking [20]. More research is needed therefore to unravel the complex dynamics of hysterectomy in India (and elsewhere) which could be used to help women make more informed choices and in turn advance their reproductive health and rights.

## Policy relevance

This study has significant policy relevance. The Indian Government is in the midst of designing and implementing a new national policy for women. The Draft National Policy for Women (NPW) 2016 focuses on, among other things, improving women’s health through a holistic and life-cycle approach that includes provision of appropriate, affordable and quality health care services. Moreover, this draft policy also explicitly recognizes the dearth of health care services for older, menopausal women [53]. The findings of this study may be used to advance women NPW 2016 objectives. Firstly, while the reason why a significant number of young women are undergoing hysterectomy may be complex, lack of education influencing hysterectomy indicates that there is perhaps a need for counselling and education on alternative options for them to be able to make informed choices. Secondly, while government health insurance schemes serve to protect the poor and vulnerable populations from catastrophic health expenditure, the evidence that it could also lead to unnecessary hysterotomies being performed warrants a need to better design health protection plans. Finally, there is need for national level health statistics and surveys, such as nationwide National Family Health Survey, to provide information on hysterectomy. Moreover, it would also be useful to design data systems that gather information on oophorectomy as well in order understand the epidemiology of hysterectomy more fully.

## Abbreviations

CAPI: Computer Assisted Personal Interviews
CI: Confidence Interval
DLHS: District Level Household and Facility Survey
EAG: Empowered Action Group of States
IIPS: International Institute for Population Sciences
NFHS: National Family Health Survey
OR: Odds Ratio
